# Neuromodulatory Treatments for Alcohol Use Disorder: A Review

**DOI:** 10.3390/brainsci8060095

**Published:** 2018-05-28

**Authors:** Anne-Mary N. Salib, Allen L. Ho, Eric S. Sussman, Arjun V. Pendharkar, Casey H. Halpern

**Affiliations:** Department of Neurosurgery, Stanford University School of Medicine, 300 Pasteur Drive, Edwards Bldg./R-227, Stanford, CA 94305, USA; asalib.ucsandiego@gmail.com (A.-M.N.S.); allenlho@gmail.com (A.L.H.); esussman@stanford.edu (E.S.S.); apendhar@stanford.edu (A.V.P.)

**Keywords:** nucleus accumbens, alcoholism, binge drinking, deep brain stimulation, neuromodulation, mouse models

## Abstract

Alcohol use disorder (AUD) is a prevalent condition characterized by chronic alcohol-seeking behaviors and has become a significant economic burden with global ramifications on public health. While numerous treatment options are available for AUD, many are unable to sustain long-term sobriety. The nucleus accumbens (NAcc) upholds an integral role in mediating reward behavior and has been implicated as a potential target for deep brain stimulation (DBS) in the context of AUD. DBS is empirically thought to disrupt pathological neuronal synchrony, a hallmark of binge behavior. Pre-clinical animal models and pilot human clinical studies utilizing DBS for the treatment of AUD have shown promise for reducing alcohol-related cravings and prolonging abstinence. In this review, we outline the various interventions available for AUD, and the translational potential DBS has to modulate functionality of the NAcc as a treatment for AUD.

## 1. Introduction

Alcohol abuse is a growing epidemic that has both public health and economic concerns. Alcohol is the fourth leading cause of preventable death in the United States [1]. An estimated 88,000 people (~62,000 men, ~26,000 women) die from alcohol-related causes annually [1]. In 2015, 26.9 percent of people ages 18 or older reported that they engaged in binge drinking in the past month [2]. In 2010, the U.S. spent an estimated $249 billion in the mitigation of alcohol misuse, three quarters of which was due to binge drinking [3]. Alcoholism is also a growing global burden. In 2012, 3.3 million (5.9%) of global deaths were attributed to alcohol consumption [4]. The staggering prevalence and repercussions of alcohol abuse has made alcoholism a public health obligation. 

Medical treatment for alcoholism involves two phases. The first phase is acute detoxification and withdrawal, and the second phase is the chronic long-term maintenance of abstinence, or the relapse prevention phase. Each phase can rely on additional pharmaceutical support to habituate addicts to sobriety less abruptly [5]. During the acute detoxification phase, benzodiazepines are used to treat the symptoms associated with withdrawal. For an addict, benzodiazepine dependence is a possible adverse outcome of detoxification treatment. 

FDA approved pharmaceutical interventions often used in the post-withdrawal maintenance of alcohol abstinence phase include medications such as disulfiram, naltrexone (NTX), and acamprosate (ACAMP). Each of these medications targets a specific component of relapse [6]. Disulfiram is a form of aversion therapy that negatively reinforces aversion toward alcohol. It inhibits the enzyme acetaldehyde dehydrogenase leading to an accumulation of acetaldehyde in the presence of ethanol, which causes physical adverse symptoms of veisalgia. Naltrexone is an opioid receptor antagonist that reduces heavy drinking by targeting and blocking the rewarding neurobiological effect of alcohol. Acamprosate is thought to normalize alcohol disrupted glutamatergic excitation that occurs during alcohol withdrawal. It targets the chemical imbalance caused by alcohol abuse by acting as GABA agonist and neuromodulator of glutamate *N*-methyl-d-aspartate (NMDA) activity [6,7]. Disulfiram and naltrexone are metabolized by the liver and can cause hepatotoxicity. The absence of liver metabolism and pharmacokinetic interactions with alcohol makes acamprosate a more viable option for patients with liver disease [8]. 

In addition to pharmaceutical support, psychotherapeutic modalities such as cognitive behavioral therapy, interventional sessions, social skills training, and motivational enhancements can be administered in conjunction with pharmacological interventions. However, none of these behavioral interventions have been found to be more effective than any other [6,9,10]. The challenge that both pharmaceutical and behavioral interventions pose is that they are heavily reliant on the consistent compliance of the patient. Initiative driven therapies are challenged by unremitting cravings, external stressors, and withdrawal symptoms. This is a major factor contributing to high relapse rates after standard pharmacological and psychotherapeutic interventions, and why alternate methods of treatment should be explored for alcoholism refractory to standard treatment. 

Deep Brain Stimulation (DBS) has been proposed as an alternative treatment for addiction with some preliminary promise of efficacy [6,11,12]. DBS has evolved over the past 30 years, from initial use for Parkinson’s Disease (PD) and other movement disorders, to more recent off-label pilot studies for treatment of neuropsychiatric disorders such as major depression, obsessive compulsive disorder (OCD), and substance abuse [11]. DBS is an attractive alternate intervention for addiction because of its on-off reversibility, relatively low complication rate, adjustability, preliminary efficacy in pilot studies for conditions such as OCD, and reduced reliance on individual initiative for compliance [13,14,15]. Clinically, DBS mimics ablation, indicating its potential for modulating specific brain regions dysregulated in a number of neuropsychiatric conditions [13]. Although the exact mechanism is not fully understood, DBS appears to work on a systems level, increasing spontaneous firing of neuronal populations and axonal projections proximal to the electrodes. However, DBS generates system effects via the back firing of afferent neurons and the activation of the projection pathways stemming from the stimulated nucleus throughout the connected network [16]. With the exception of minor gliosis around the electrodes, permanent implantation has no further side effects nor histopathological damage to the surrounding tissue [17,18,19]. While the electrode placement would be permanent, treatment reversibility involves simply turning off stimulation if unwanted effects are observed. Medtronic Inc. developed the first FDA-approved DBS device. One hundred and forty-five thousand individuals have undergone implantation worldwide, 135,000 of which have received Medtronic DBS Therapy for Parkinson’s Disease (http://www.medtronicdbs.com).

**Figure 1:**
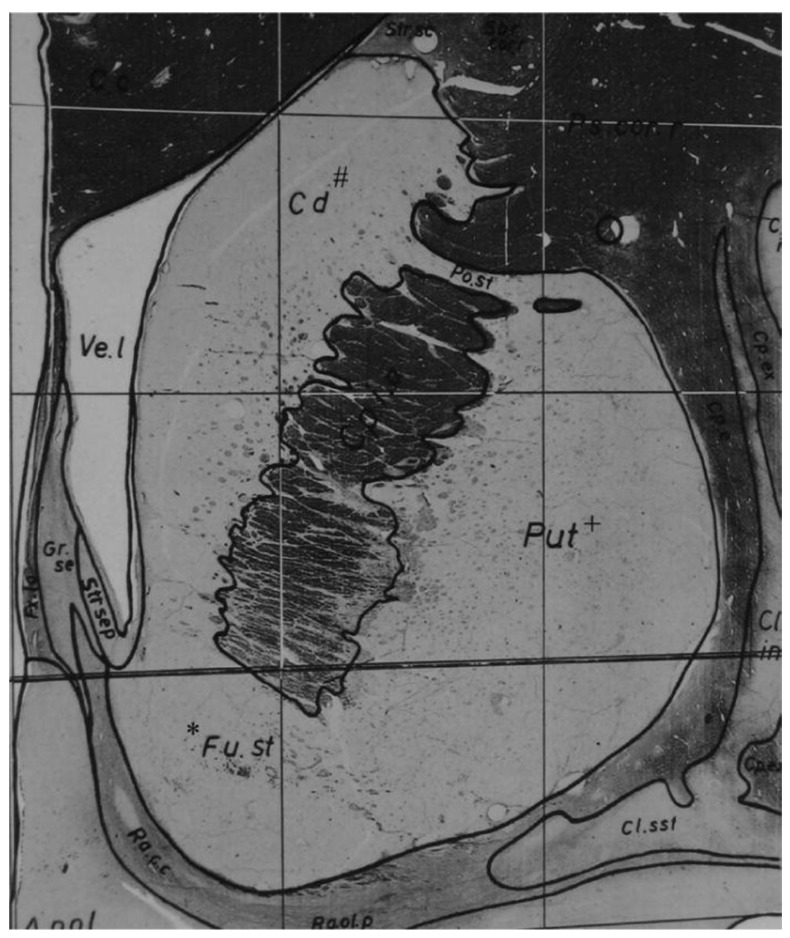
Nucleus accumbens. A coronal human tissue section from the Schaltenbrand G, Warren W: Atlas for Stereotaxy of the Human Brain depicting the nucleus accumbens located in the ventral striatum just inferior to the caudate nucleus and inferolateral to the lateral ventricle. * Nucleus accumbens (Fu.st); # caudate nucleus (Cd); + putamen (Put). Adapted from [23], permission obtained.

**Figure 2:**
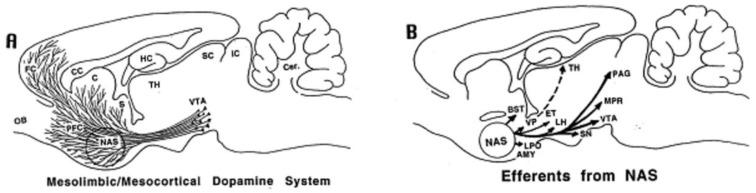

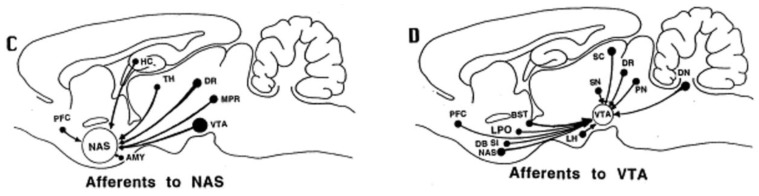
Mesocorticolimbic reward circuitry of the rat brain. (**A**) Ascending projections of dopamine (DA) neurons (localized in the VTA) innervating to limbic regions including the nucleus accumbens (NAS, the mesolimbic DA system) as well as cortical regions (the mesocortical DA system); (**B**) Major efferent projections of the NAS; (**C**) Afferent projections to the NAS; (**D**) Afferent projections to the VTA. Abbreviations—AMY, amygdala; BST, bed nucleus of stria terminalis; C, caudate–putamen; CC, corpus callosum; DA, dopamine; DB, diagonal band of Broca; DN, dentate nucleus; DR, dorsal raphe; ET, entopeduncular nucleus; FC, frontal cortex; HC, hippocampus; IC, inferior colliculus; LH, lateral hypothalamus; LPO, lateral preoptic area; MPR, mesopontine reticular nuclei; OB, olfactory bulb; PAG, periaqueductal gray; PFC, prefrontal cortex; PN, parabrachial nucleus; SC, superior colliculus; SI, substantia innominata; SN, substantia nigra; TH, thalamus; VP, ventral pallidum. Adapted from [27], permissions obtained.

The nucleus accumbens (NAcc) is a grey matter substructure of the ventral striatum that upholds a central role in the mesocorticolimbic reward circuitry. (Figure 1) As a primary input structure of the basal ganglia, the NAcc has implications of establishing and mediating the rewarding sensations elicited by both natural rewards and drugs of abuse [20]. Ethanol produces its reinforcing action through the midbrain dopaminergic system which includes mesocorticolimbic projections from dopaminergic neurons in the VTA to the NAcc, amygdala, and frontal cortex [21,22]. (Figure 2) The sub-territory of the NAcc, referred to as the shell has been identified as a critical component for the self-administration of drugs of abuse [7,21]. Molecularly, the NAcc core and shell differ in the concentrations of dopamine and serotonin [23]. Amine ratios suggest that both dopamine and serotonin utilization are greater in the NAcc shell in comparison so the NAcc core [23].The NAcc shell contains a larger number of dopamine receptors, while the core contains more dopamine transporters [23,24]. Opiate drugs and ethanol increase extracellular dopamine in the shell more than in the core [25,26].

## 2. Mouse Models

A diagnosis of Alcohol Use Disorder (AUD) based on DSM-V is determined by the presence of at least two of eleven possible criteria listed within the past year [28]. This broad range of categorization creates immense heterogeneity in the clinical and etiological manifestations of this disorder. Because of the diversity of AUD, no single animal model can represent all the various combinations and complexities embodied in human alcoholism. However, across multiple partial paradigms it is possible to capture hallmarks of analogous physiology and behaviors. The legal, economic, and social ramifications contributing to the stress and overall impulsivity of compulsive alcohol consumption is difficult to reproduce in animal models. 

A commonly utilized preclinical mouse model depicting alcoholism is preference drinking involving a two-bottle choice that entails providing animals with access to two bottles: one with water and the other with varying dilutions of ethanol in water. The animal’s genotype can strongly influence ethanol preference and self-administration, with different lines bred to be high or low in alcohol preference [29]. The National Institute on Alcohol Abuse and Alcoholism (NIAAA) define a ‘binge’ as a pattern of drinking that produces blood ethanol concentrations (BECs) of greater than 0.08% (80 mg/dL) [9]. For mice, a binge is defined as 100 mg/dL because of their high rate of ethanol metabolism [30]. To model heavy drinking, genetic strains including C57BL6 and C57BL/6J were identified as genetically predisposed to high alcohol preference (HAP), while strain DBA/2 was bred to model low alcohol preference (LAP) [31]. HAP mice also tend to consume more saccharin than LAP mice, consistent with the correlation between sweet preference and alcohol drinking [32]. For this reason, sucrose fading is an effective method to initiate and maintain ethanol self-administration. Briefly, sucrose fading is a stimulus substitution technique that involves an initial adulterated sucrose ethanol mixture, which over time is adjusted such that the ethanol concentration of the mixture would gradually increase while sucrose decreased until eventually only ethanol was presented as the reinforcing stimulus [33]. Because HAP animal models tend to consume more saccharin and ethanol, it can be complicated to characterize binge behavior specific to ethanol when utilizing sucrose fading. 

A challenge for developing a representative animal model for craving or relapse is the issue of ethanol dependence history. With the exception of animals genetically selected for HAP, most animals will not voluntarily consume ethanol at levels sufficient to induce dependence [34]. The Alcohol Deprivation Effect (ADE) is a model for relapse that entails a marked increase in ethanol consumption following periods of alcohol deprivation approximate to relapse in otherwise abstinent alcoholics [35]. ADE is most robust in the two-bottle free choice procedure using genetically bred alcohol-preferring animals or after extensive repeated cycles of intoxication and deprivation [36]. Given the importance of rodent behavioral models for studying the role of the NAcc in AUD, especially with neuromodulatory interventions, we will highlight and review two of the more popular and robust rodent AUD models.

## 3. Drinking in the Dark (DID)

To induce pharmacologically significant ethanol drinking in mice, Rhodes et al. [37] tested multiple paradigms involving intermittent access to ethanol in various time points in the sleep-wake cycle. Using C57BL6 male and female mice, on a 12:12 light:dark cycle (22:00 lights off, 10:00 lights on), mice drank approximately 90% more ethanol in a 2-h period when ethanol was substituted for water starting 3 h after lights off (active phase of circadian cycle) in comparison to starting 1 h after lights off. Similarly, mice drank 50% more when starting 3 h after lights off in comparison to 2 h after lights off [37]. This procedure yielded the animal model referred to as “drinking in the dark” (DID), through the promotion of high levels of ethanol drinking–mimicking that of binging, without the need of extensive training or the inclusion of sucrose. By replacing the water bottle with 20% ethanol for 2 or 4 h in the mouse’s home cage, starting 3 h after lights off, and repeating across four consecutive days, mice reliably drank ethanol to levels that produce a BEC of 1.0 mg/mL by day 4. 

There are several unique advantages to the DID system in modeling binge-like ethanol consumption. First, it reliably achieves pharmacologically meaningful BECs in a well-defined, limited access time frame [30]. This allows for ease of experimental design for high throughput testing of the effects of pharmacological compounds or other more invasive interventions on binge-like ethanol intake, because the ethanol access occurs over a relatively truncated period of time [37]. Indeed, repeated episodes of DID procedures leads to relatively consistent binge-like ethanol drinking, even up to 10 repeated 4-day DID procedures [38]. Exhibition of binge-like drinking behavior with short-term ethanol access more precisely mirrors human binge drinking behavior. In other ethanol binge models where ethanol is directly administered by the experimenter, these passive, forced, or potentially painful methods of ethanol administration can confound the true motivation to binge ethanol [21]. The DID paradigm allows for preclinical modeling of binge drinking, a hallmark of AUD, in the context of potential therapeutics, such as DBS. 

## 4. Intermittent Access (IA)

A series of experiments conducted by Simms et al. were designed to further characterize intermittent access to 20% ethanol using 2-bottle choice drinking paradigms. The experiments were adaptations from Wise [39], in an effort to induce ethanol consumption without adulteration. Animals were given access to 20% ethanol for three 24 h sessions per week. Ethanol access was intermittent in the sense that access was reinstated every other day. Using a two-bottle choice, animals had continuous access to at least one bottle of water at all times. However, the second bottle alternated between 20% ethanol and water. In experiment one, animals were given access to 20 ethanol-drinking sessions (across 45 days), water only and ethanol + water consumption was recorded. This resulted in a steady escalation in ethanol intake until the fifth drinking session, thereafter, there was no difference in ethanol consumption in subsequent sessions. Experiment two involved a 40-day period of abstinence following the 20th ethanol drinking session using the rats from experiment one. Following the abstinence period reinstatement of ethanol using IA was conducted across ten drinking sessions. There was no significant difference in mean ethanol consumption (g/kg/24 h) between the ten drinking sessions before and after the abstinence period [40]. Preclinical models utilizing both IA and DID allow for unadulterated consumption of ethanol that better represent the pathophysiology of AUD.

## 5. Disrupting Binge Behavior in Animal Models

There have been several successful pharmacologic and surgical interventions geared towards attenuating binge-like ethanol consumption in animal models. Pharmacological inactivation through the utilization of a neuronal inhibitory agent such as a GABA agonist clinically emulates an anatomic inhibition similar to DBS, enabling further investigations of the role of specific anatomical regions in the context of a behavior. In female rodent models, bilateral pharmacological silencing of the NAcc shell with GABA agonists baclofen and muscimol did not decrease the acquisition (lever presses) of ethanol (EtOH) drinking behavior, but did reduce EtOH consumption in chronically drinking rats. While, 200 μA of DBS also consistently reduced EtOH consumption in chronically drinking rats. The amount of EtOH consumption returned to baseline levels following termination of therapy in both paradigms [41].

NTX and ACAMP neurochemically target different components of alcohol seeking behavior. NTX is a µ-opioid receptor antagonist while ACAMP is a NMDA receptor antagonist. Through the alcohol deprivation effect, ethanol seeking increases after a period of imposed deprivation. This increase in ethanol seeking was pharmacologically blocked by chronic administration of ACAMP in rodents [36]. The effect of NTX and the combination of NTX + ACAMP on oral ethanol self-administration was also observed following an imposed period of abstinence. In a two-lever free-choice ethanol self-administration experimental design where rats were conditioned to access ethanol or water via different lever pushes, response for ethanol reinstatement increased significantly following a deprivation period. The NTX only group and the combination NTX + ACAMP blocked the increased ethanol consumption following the abstinence period on post-deprivation day one. On day two, the combination of ACAMP + NTX group sustained the blocked increase in ethanol consumption, while the NXT group alone did not [42]. Thus, while both NTX and ACAMP seem to quell acute ethanol seeking following periods of deprivation, ACAMP may be more effective at sustained consumption amelioration than NTX alone.

## 6. Deep Brain Stimulation

Rodents undergoing stimulation of the NAcc demonstrated a decrease in consumption and preference when self-administering high-fat chow, ethanol, cocaine, and morphine but not water or regular chow [12,43,44]. This is thought to be due to alterations of dopamine physiology with DBS [15]. Thus, NAcc stimulation mimics the effects of a lesion, and can be thought of in the same way as local dopamine depleting/inhibitor injections such as baclofen and muscimol [45,46]. Long-term structural disruptions via lesioning the NAcc shell reduced alcohol preference in HAP rats [15], and abnormal dopaminergic transmission to NAcc has been implicated as a potential mechanisms of DBS in the context of addiction [44].

One of the first studies of DBS for ethanol consumption was completed by Henderson et al. in 2010. Male rats had ad libitum access to food, water, and 10% ethanol for three to seven weeks to establish baseline levels of alcohol consumption. Bilateral DBS electrodes were placed in the outer shell of the NAcc. Specific locations of the stimulating electrodes were calculated from the bregma and brain surface using stereotactic coordinates: anteroposterior +1 mm, mediolateral ±3 mm, and dorsoventral −8 mm [47]. Post-surgery animals were moved to operant chambers and continued on the same ad libitum regimen for ethanol and water access. Animals underwent DBS for two days, using 2 h treatment blocks (1 h on, 1 h off). Animals were randomly assigned stimulation in the first hour or the second hour of the two-hour block on the first day of treatment, and the treatment order was reversed on the next day. This set up made it so each animal served as its own control. Alcohol was withheld from rats until the start of the dark cycle, and then reintroduced. Stimulation parameters consisted of monophasic square wave pulses with a frequency of 140–150 Hz with a pulse width of 60 μs and a current intensity of 200 μA. Alcohol consumption decreased approximately 30% during periods of DBS in the NAcc as compared with the non-stimulation study periods in the same animals, however, this decrease was deemed as statistically insignificant [44]. 

A second cohort of animals were given access to alcohol for four to six weeks to establish baseline levels of consumption. Access was then discontinued for four to six weeks. During abstinence, electrodes were implanted. Animals were randomly assigned to a sham control or treatment DBS group. While animals in the DBS treatment group received DBS with the same parameters as the first cohort, they were allowed free access ethanol, water, and food for 24 h. Both alcohol consumption and preference over the 24 h exposure period were significantly reduced in the DBS treatment group versus the control sham group indicating attenuation of ADE in HAP rats [44]. The increased ethanol exposure time in this second cohort of mice, compared to first, accounts for the more obvious differential effect seen with DBS. This is supported by evidence from both DID and ID models discussed above that have revealed binge behavior that occurs optimally for several hours at a time. 

A more recent preclinical animal model sought to understand the effects of chronic bilateral deep brain stimulation of the NAcc on gating the relapse-like drinking behavior (ADE) in rat models [48]. To determine the overall network effects of DBS in alcohol-dependent rats, this group combined stimulation of the NAcc with functional magnetic resonance imaging (fMRI) data, and neurotransmitter (DA) levels in the NAcc in stimulated versus sham rats. DBS electrodes were implanted bilaterally into the NAcc shell (NAccs), caudate putamen (CPu), and infralimbic (IL) cortex. DBS was applied at 130 Hz, 5.25 V, and 90 μs pulse duration. Electrical stimulation-functional Magnetic Resonance Imaging (es-fMRI) using a horizontal 7T scanner was utilized post electrode implantation to obtain T2-weighted images. For stimulation during fMRI recordings, charge balanced biphasic 0.1-ms duration pulses of 150 μA with 90 μs pulse duration were delivered at 130 Hz. The protocol consisted of ten repetitions of stimulation ON periods lasting for eight seconds, followed by OFF periods of 26 s. 

Animals were subjected to chronic continuous DBS, three days prior to alcohol reinstatement, and es-fMRI data revealed activation in the medial prefrontal cortex and caudate putamen. However, stimulating these areas did not affect relapse behavior. Neurochemical analysis comprised of tissue high performance liquid chromatography (HPLC) and in vivo microdialysis of these activated brain areas revealed that the effect of stimulation may rely on NAcc dopamine levels, given that the rats that underwent DBS exhibited augmented alcohol-induced dopamine release compared with sham-stimulated animals. Based on results of this study, the NAcc may also play a role in enhancing alcohol addiction via augmented dopamine release and can thereby promote relapse [48].

## 7. Clinical Trial Data

The DSM-IV categorizes alcohol misuse into two distinct diagnoses, alcohol abuse and alcohol dependence [49]. The DSM-V, integrates both alcohol abuse and dependence into one disorder called alcohol use disorder (AUD). AUD is a chronic relapsing brain disorder characterized by compulsive alcohol use, loss of control over alcohol intake, and a negative emotional state when refraining from alcohol. Anyone meeting any two of the eleven criteria outlined in Table 1 during the same 12-month period receives a diagnosis of AUD [28]. The total number of criteria met determines the severity of AUD—mild, moderate, or severe [28]. Binge drinking is a component of AUD, defined as drinking that brings the blood alcohol concentration (BAC) levels to 0.08 g/dL in about 2 h [4]. As alcoholism continues to be a global concern, the International Statistical Classification of Diseases and Related Health Problems [50] is also a means of determining international and statistical classifications of disease related health problems including alcohol abuse disorders sub-classified to include comorbidities found with alcohol use and dependence. A variety of pilot clinical trials have been designed and executed to attempt to treat alcohol use disorder. All, to some degree, have failed to address the multifaceted nature of alcohol addiction, particularly in achieving long-term abstinence. In particular, the human DBS data presented below represents preliminary case reports and a single small pilot study that should prompt future controlled investigation. 

## 8. Baclofen Trial

A study conducted by de Beaurepaire in 2012 intended to study the long-term effects of baclofen in 100 patients with alcohol dependence resistant to conventional treatments. Baclofen demonstrated limited efficacy for patients with comorbid mental disorders, concurrent use of psychotropic drugs, and “lack of real motivation to stop drinking” which could be in part due to the inability to reach the optimal dose—because of the intolerability of side effects. Fifty-nine percent of the participants had one of several psychiatric disorders including anxiety, depression, bipolar, psychosis, sleep disorder, eating disorder, and other substance abuse, and 88% reported at least one undesirable side-effect while on baclofen [51,52]. Thus, baclofen therapy is limited in the AUD population due to its side effect profile at therapeutically effective doses and the high proportion of comorbid psychiatric disorders in the AUD population that preclude its use.

## 9. Transcranial Direct Current Stimulation (tDCS)

In 2013, Da Silva et al. [53] conducted a sham-controlled study that targeted unilateral treatment of the frontal lobe dysfunction with the intent of reducing cravings and relapse through the use of repetitive unilateral anodal stimulation of the left-dorsolateral prefrontal cortex (L-dlPFC) in 13 heavy drinkers (200 g ethanol a day). Initially, participants who received the L-dlPFC treatments were found to have significant decrease in craving and depressive symptoms, as well as increased executive function in comparison to the sham group. However, these results were short lived because 4 of the 6 in the treatment group relapsed during the trial, compared to 1 relapse in the sham group [53]. Later, in 2014, Klauss et al. [54] conducted a trial of bilateral tDCS stimulation of the dlPFC in 35 severe alcoholics that were randomized into sham and treatment groups in the study. Two of the 16 in the sham group, and 8 in the treatment group remained alcohol abstinent by the end of the six months of follow-up. No differences were reported with regards to cravings, frontal function, and depressive/anxiety symptoms [54]. Modest effects seen with tDCS on cravings are tempered by high relapse rates and long-term efficacy for this therapy remains to be established.

## 10. Repetitive Transcranial Magnetic Stimulation (rTMS)

In 2010, Mishra et al. conducted a trial involving rTMS of the right dorsolateral prefrontal cortex (R-dlPFC) in 45 patients diagnosed with alcohol dependence syndrome. 30 participants received active rTMS treatment, and 15 received sham treatment. Within the first 4 weeks following rTMS treatment-cravings significantly decreased in active treatment group, however, beyond 4 weeks there was no significant difference between the treatment and sham groups with regards to cravings. Similarly, there was no significant difference between relapse rates between the two groups [55]. In a follow-up study in 2011, Hoppner et al. [56] conducted a trial involving rTMS of the left dorsolateral prefrontal cortex (L-dlPFC) in 19 detoxified females. The intent was to investigate the effect of rTMS on mood and cravings in alcohol-dependent women. There were no significant differences between the treatment and sham groups with regards to mood or cravings [56]. Again, while at best a temporary decrease in cravings was seen with rTMS therapy, this was not durable in the long term and had no effect on relapse rates.

## 11. Disulfiram Intervention

Ethanol is converted to acetaldehyde by alcohol dehydrogenase, and acetaldehyde is then converted to acetate by aldehyde dehydrogenase. Disulfiram blocks enzyme aldehyde dehydrogenase and is a common pharmacological intervention in the treatment of alcohol dependence. In the presence of alcohol, acetaldehyde accumulates to cause symptoms including tachycardia, flushing, diaphoresis, dyspnea, nausea, and vomiting creating a deterrent to alcohol ingestion in a controlled environment. However, efficacy of disulfiram drops tremendously in an unsupervised environment as compliance with the regimen and succumbing to relapse becomes a decision at the discretion of the patient [57]. A longitudinal trial involved 120 alcoholic volunteers who were assigned at random to receive subcutaneous disulfiram implants and were followed for a subsequent 2-year period. Post-implantation all groups demonstrated an increase in sobriety, however, there was no significant dose response relationship nor was there a significant difference between groups in the incidence of disulfiram ethanol reaction [58]. 

## 12. 2007 DBS Case Study

A case study of a 54-year-old male patient with severe anxiety disorder, secondary depressive disorder, and long term heavy alcohol dependence underwent bilateral DBS [14]. The patient’s initial labs revealed a carbohydrate deficient transferrin (CDT) value of 5.2% and gamma-glutamyl transferase (GGT) of 185 U/l, indicative of metabolic dysfunction consistent with severe alcoholism. The justification behind this pilot trial was based on the severity of the symptoms, the refractory nature of the psychiatric disorder in this individual, the reciprocated interest of the patient, and the efficacy DBS had in the past in the context of treating OCD and anxiety [14]. A previous pilot series by Sturm et al. had demonstrated effective results with NAcc stimulation for severe OCD and anxiety based off the well-established pathophysiology of the NAcc associated with anxiety disorders and reward circuitry within the amygdaloid complex, basal ganglia, and mediodorsal thalamic nucleus and prefrontal cortex [14,59,60].

Quadripolar electrodes were bilaterally implanted using a deep frontolateral approach, 4.5 cm rostral of the coronal suture and 4.5 cm lateral left and right of the mid-sagittal suture. Electrode placement was determined by an individualized image fusion of MRI and intraoperative CT. Three-dimensional coordinates for the target site were 1 mm rostral anterior border of anterior cingulate, 7 mm lateral of midline, and 4 mm ventral anterior cingulate. (contact 0,1: fundus subventricularis medialis of the nucleus accumbens; contact 2,3: anterior limb of the internal capsule). A pulse generator connected to the electrodes was implanted infraclavicularly. Post-operation adjustments to stimulation parameters were tested for efficacy and adverse reactions. The optimal settings consisted of bilateral 0, off; 1, −; 2, −; 3, off; case +; pulse width 90 µs, 130 Hz, 3 V. During the 12 month treatment period, the applied voltage was increased to 4.5 V [14].

Post-stimulation, the patient reported pleasant acute feelings of “inner appeasement”. However, he demonstrated little to no reduction of anxiety disorder or depressive mood symptoms following DBS. Interestingly, the patient did drastically and rapidly reduce alcohol consumption without being cued or instructed to do so. Twelve months post-DBS, the patient reported a loss of desire to drink alcohol, coupled with a reduction in overall alcohol consumption. His GGT and CDT values normalized to 43 U/l and 1.5% respectively, which substantiated reduced alcohol consumption [14].

## 13. 2008–2016 DBS Pilot Trial

A study conducted in Germany consisted of 5 patients with treatment-resistant alcohol dependency (based on DSM4 and ICD-10 criteria) that underwent DBS treatment and longitudinal follow-up care beginning in 2008. The inclusion criteria for this study included males, ages 25–60, with 10 years or more of alcohol dependence, who completed detoxification and subsequent period(s) of abstinence for at least 2 weeks. To be eligible for this study, participants had to have demonstrated treatment failures of at least two inpatient programs (6 months or longer for each program), failure of anti-craving pharmaceutical interventions such as ACAMP and NTX, as well as failure of community and self-help intervention programs. Participants had to have had at least 10 years of schooling in addition to a “finished apprenticeship or higher education” to ensure they had the mental capacity to perform psychological tests before and after implantation and understood the procedures and associated risks. Patients included in this study were considered severely addicted to alcohol and, on average, consumed at least 200 g of ethanol a day [6,61,62].

Participants were excluded if they had any of the following: seizures during detoxification, mental retardation, clinically significant impairments on neuropsychological battery, alcohol related personality change, use of additional addictive substances, or marked atrophy signifying brain damage detected via MRI. Additionally, high scores on the neuroticism scale and/or antisocial personality disorder were exclusionary [61]. It is important to note that all patients were offered continuous care for their DBS device as well as psychiatric outpatient care covered by their compulsory health care insurance, making all treatment free of charge [62].

Implantation of the electrodes were localized to the ventro-caudo-medial accumbens, which required adjustments to stereotactic coordinates to account for individual anatomical variations, determined by MRI. The target was defined as 2 mm rostral to the anterior border of the anterior commissure at the level of the mid-sagittal plane, 3–4 mm ventral, and 6–8 mm lateral of the midline. The target was placed 2–2.5 mm lateral of the vertical limb of Broca’s diagonal band. Using a deep fronto-lateral approach, the two distal contacts of the DBS-electrode were placed in the caudo-medial accumbens, the third contact within the transition-area to the medial border of the internal capsule, with a fourth connection point in the most medial part of the capsule. The contacts within the NAcc were placed in the caudo-medial area [61]. 

Each patient in this trial had a unique experience with his DBS treatment. Patient 1 remained abstinent from the time of his DBS initiation in 2007 to the follow-up reported in 2016, nearly 8 years later. Patient 2 also remained abstinent since initiation of DBS in 2008 without relapsing until he was lost to follow-up in 2014. Patient 3 continued to relapse after initiation of DBS in 2007, underwent a 12-week inpatient rehab treatment, and continued to relapse after short lapses of abstinence. He accredited DBS with the elimination of his alcohol cravings, but reported significant external stressors that drove his relapses. Nearly three years after implantation he was imprisoned for almost 4 years. His alcohol cravings resurfaced after his battery ran out of power while incarcerated. The patient died soon after, and the study authors assumed that DBS had no influence on his death, but rather continuous consumption of alcohol may have contributed. Patient 4’s clinical records and lab results indicated abstinence for over 12 months following initiation of DBS. He claims he then relapsed for a few days out of curiosity. He had additional relapses with subsequent detoxifications after 17, 21, 26, and 31 months. He stated that DBS helped him to avoid relapsing continuously, and that he accredited relapses to stress, rather than cravings. His lab results indicated relapses lasted for at least one to two weeks, though he always self-reported that his relapses were of a few days length only. After 36 months, he was diagnosed with a major depressive episode that was treated into remission with an SSRI and psychotherapy. He continued to relapse during the fourth year of DBS. He claimed alcohol helped him cope with the sudden death of his healthy and sober brother, while he continued to live as an addict. He was eventually lost to follow-up and was found dead at his home. Patient 5 relapsed nearly 20 months after DBS initiation and continued to have short relapses about every 2–3 months to “relieve” negative stress, but stated that craving for alcohol had persistently disappeared. He received outpatient psychotherapy treatment at the same treatment center 24 months after initiation of DBS due to feelings of guilt and depressive symptoms. During that time, he did not remain abstinent and was unable to avoid drinking. He started antidepressant medication and depressive symptoms were alleviated. 

All 5 patients reported disappearance of cravings for alcohol, including cue-induced cravings in everyday life, consistent with the Alcohol Urge Questionnaire (AUQ) responses obtained post DBS initiation. Despite a reduction in declarative urge, select patients were unable to remain completely abstinent, leaving room for the possibility that consumption of alcohol essentially shifted from goal-directed behavior to habitual behavior. The absence of craving for alcohol is consistent with the hypothesis that DBS of the NAcc may be modulating the reward system of the brain, which was otherwise modulated maladaptively by alcohol [63,64]. Two of the five patients died with implications that continuous alcohol consumption contributed to their deaths. With the exception of one patient (patient 3) experiencing transient hypomanic symptoms directly after DBS initiation, no other adverse side effects associated with DBS were observed or reported. Stimulation parameters for individual patients were not changed throughout the course of treatment [6,61,62,65]. 

Subsequent testing with patient 3 revealed that active DBS was associated with somewhat slower and less risky choices, implying a more impulsive, riskier, and less controlled behavior when neural activity was not modulated by DBS. DBS affecting the NAcc/bed nucleus of the stria terminalis (BSTM)/ventral pallidum (VP) regions made an impact on reward processing. Behaviorally, the patient demonstrated a tendency towards more risky behavior when the stimulator was turned off. A similar impulsive behavioral pattern is known from Parkinson patients treated with drugs affecting dopaminergic D2/D3 receptors [65]. PET revealed win- and loss-related activations in the paracingulate cortex, temporal poles, precuneus, and hippocampus under active DBS, brain areas that have been implicated in action monitoring and behavioral control. With the exception of the temporal pole, these activations were not seen when DBS was deactivated [65].

## 14. Clinical Trial Challenges and Considerations

What all the studies outlined above had in common was the inability to successfully sustain long term craving suppression that translated into long term abstinence. 

There are several factors to consider when designing a clinical trial that should target and optimize the efficacy and sustainability of abstinence. As the rates of adolescent alcohol abuse increases, there might be some merit to designing studies that target early intervention. Another factor to consider is standardizing the severity and scope of alcohol abuse through the DSM and/or ICD-10. Similarly, defining the scope of relapse and sustained abstinence in terms of procedural, medical, and metabolic hallmarks. Moving forward, future trial parameters should incorporate the possible shortcomings of previously failed interventions. Criteria pertaining to age, sex, failed pharmacological or procedural interventions, and comorbid psychiatric conditions should be taken into consideration. Alcoholism is often a byproduct of psychiatric and/or behavioral issues, thus, designing a trial that is representative of the scope and range of AUD will be pivotal to sustained abstinence. It may be valuable to include behavioral testing in conjunction with metabolic panels to support self-reported data on relapse. The ideal study candidate will also need to have strong social and economic support systems in place prior to trial enrollment. Previous studies required failure of long term in-patient rehabilitation for at least 6 months or unsuccessful pharmaceutical interventions. This creates an inherent selection bias that is important to consider, as the motivation behind seeking abstinence or the resistance/commitment involved in regimen reliant protocols may be compromised in these patients. As the utility of DBS grows in a number of treatment resistant conditions, the case studies outlined have revealed promising results in the potential use of DBS in refractory cases of alcoholism. 

Further studies involving DBS for AUD may allow for refinement of stimulation parameters and identification of advantageous combinations of DBS and psychotherapy that can prevent relapse. Additional clinical and preclinical trials are necessary to inform whether the benefits of DBS for AUD outweigh the ethical concerns of the invasiveness and permanence of the procedure. The heterogeneity of AUD and the consequential medical conditions causally linked to increased alcohol consumption are important considerations when designing a trial testing the efficacy of DBS on AUD. Teasing apart which comorbidities to preclude in a population predisposed to liver disease, poor nutrition, skin infections, diminished immune response, cardiovascular disease, and psychiatric conditions poses a unique challenge for generalizing and assessing the safety profile of DBS for alcoholism. The gold standard for intervention based clinical trials are randomized, double-blind, and placebo/sham-controlled. While the feasibility of conducting such trials is conceptually simple, given the on-off reversibility of DBS, ethically, a study involving implanting all participants, and administering stimulation to a subset of them, would call to question whether withholding active treatment from participants warrants the surgical risks.

Staggering the onset of active treatment may help remedy this concern, enabling all participants access to active treatment at some point in the trial. The first double-blind, sham controlled trial testing bilateral stimulation of the NAcc for treatment refractory OCD consisted of three sequential treatment phases to remedy these ethical concerns [17]. After electrode implantation, 16 participants entered an open phase of 8 months which involved biweekly evaluations for severity of symptoms and optimization of stimulation parameters. Approximately two months after implantation, a standardized cognitive behavioral therapy (CBT) program was added to the participant intervention protocol, and continued throughout all phases of the study. After the open phase, 14 of the 16 participants entered a month long, double-blind, sham-controlled phase. Participants were randomly assigned via computerized block randomization to a month consisting of 2 weeks of active stimulation and two weeks of sham stimulation. Patients were assessed at baseline, after a 2-week period of active or sham stimulation, and after the second 2-week period of reversed active or sham stimulation. This allowed for each participant to serve as their own control [17]. In this pilot OCD trial, nine out of sixteen participants responded and seven out of sixteen did not, but investigators concluded that bilateral deep brain stimulation of the NAcc may be an effective and safe treatment for treatment-refractory OCD. DBS is now an approved treatment for OCD in Europe and carries a humanitarian device exemption (HDE) for use in the USA. It is worth noting that the duration of follow up in this and other DBS trials of psychiatric conditions involves a 12-month follow-up period. Judged by this criteria, the Muller et al. pilot study presented would have had 4 out of 5 participants in remission. 

The “catch 22” situation for AUD clinical trials is that of early intervention and stringent inclusion criteria for deeming a patient as treatment resistant. This is especially true for the increasing number of adolescents diagnosed with AUD. It may be that patients in earlier stages of AUD may respond better to treatments like DBS, but the cohorts evaluated thus far, are those with severe and long-standing addiction. In the Muller et al. trial, individuals were consuming 200 g of ethanol a day, which translates to about 14 standard drinks (https://www.niaaa.nih.gov/alcohol-health), over the course of 17–23 years prior to DBS intervention [6,17,61,62]. Additional trials with intermediary durations of addiction in the context of treatment resistance may result in longer remission periods. As the utility of DBS expands to more vulnerable populations with treatment-resistant conditions, there may be benefits to designing trials that incorporate an informant or study partner to ensure comprehension of risks and benefits, support participant transition to remission, and provide an alternate perspective regarding patient progress beyond self-reported data [17]. Additional considerations regarding the frequency and nature of follow up care and DBS aftercare including but limited to battery replacement, combined behavioral interventions, and medical management of comorbid conditions. 

## 15. Cost Effectiveness

The economic burden excessive alcohol use poses consists of healthcare costs, loss of workplace productivity, criminal justice, and motor vehicle crashes. The average annual cost varies from state to state, with a wide variability. According to the CDC, California incurs $35 billion dollars worth of alcohol misuse costs. The estimated total cost of excessive drinking in 2010 was $249 billion, $19 billion of which was due to binge drinking. Government costs associated with alcohol misuse in that same year was $100.7 billion, $78.7 billion of which was accounted for by binge drinking [3].

The COMBINE study was designed to evaluate efficacy of medical and behavioral therapies in the context of alcohol dependence and cost. The trial consisted of 1383 participants with a diagnosis of primary alcohol dependence according to DSM-IV criteria [66]. Participants were assigned to one of nine treatment arms involving various combinations of pharmacological interventions (NTX, ACAMP) in conjunction with medical management (MM) or combined behavioral intervention (CBI) across 16 weeks of treatment. The cost of each COMBINE intervention was determined based on the sum of medication, labor, space, and laboratory costs for each treatment condition. Focusing just on effectiveness, MM + naltrexone + acamprosate was not significantly better than MM + naltrexone. Based on the mean values of cost and effectiveness, 3 interventions were cost-effective options relative to the other interventions for all three outcomes: (MM) + placebo ($409 cost per patient), MM + NTX ($671 cost per patient), and MM + NTX + ACAMP ($1003 cost per patient). An estimated 45–75% of alcoholics that undergo treatment will relapse within 3 years, posing a long-term perpetuation of economic repercussions [66]. 

Given the chronicity and refractory nature alcohol use disorder often poses, DBS may have cost advantages associated with the potential efficacy, reversibility, adjustability, and durability. DBS has well-established efficacy and safety in the context of movement disorders; with motor score improvements as high as 66% [13,67]. For DBS, using Medicare reimbursement as a proxy for societal costs resulted in a total of $15,892.75 for the average patient [16,68,69] Private insurer and out-of-pocket expenses may be significantly higher, and long-term follow-up costs including battery replacement must also be considered. Case studies have indicated promising prolongation of the duration abstinence [11,61,70]. Preclinical studies have supported the neuromodulatory potential of DBS of the NAcc for alcoholism, as well as clinical case reports and case series of NAcc DBS that have subsequently demonstrated promise in controlling cravings and risk of relapse in both alcoholism and smoking [11,61,70,71]. However, no clinical trial to date has been performed in the United States to investigate the potential of NAcc DBS as a therapeutic alternative for alcohol dependence. Studies of DBS for alcohol use disorder and binge drinking are currently under investigation. A comparison reference of untreated (or relapsed) heroin dependence costs society more than $100,000 for a 6-month period. The cost for the average 6-month methadone (MMT) course of treatment is estimated at nearly $58,000. For patients who complete a course of MMT and are heroin-free, the total cost is limited to that of undergoing MMT. Patients who relapse have the additional cost associated with their aftercare. DBS has the potential to prolong the duration of abstinence while alleviating the expectation of continuous compliance from patients.

## 16. Conclusions

The multifaceted nature of AUD has introduced layers of complexity in the development of targeted treatments. Neuromodulation of the NAcc for the treatment of AUD has shown preliminary promise in the reduction of cravings and prolonged remission. Additional studies are necessary to further substantiate efficacy, with an emphasis on mitigating outside psychosocial stressors encountered during treatment periods. Future efforts may also inform the underlying molecular and electrophysiological signatures that hallmark the pathologic progression of AUD from impulsively goal-driven to compulsive habitual behavior. As the nuances of AUD are physiologically identified, neuromodulatory technologies can be leveraged to provide refined treatments.

## Figures and Tables

**Table 1:** Patient Summary from Pilot Trial.

Patient Number	Age at Initiation of DBS	AUQ before DBS	AUQ after DBS	Rechargeable DBS Generator	Years Addicted
1	40 (2007)	37	8	2012	23
2	35 (2008)	29	8	Unknown	17
3 *	37 (2007)	53	8	2014	22
4	51 (2008)	20	8	N/A	21
5	55 (2009)	14	8	N/A	19

* Patient 3 was the only patient that experienced a stimulation related side effect (transient hypomanic symptoms). AUD—Alcohol Urge Questionnaire.
